# Why Do Children Engage in Sedentary Behavior? Child- and Parent-Perceived Determinants

**DOI:** 10.3390/ijerph14070671

**Published:** 2017-06-22

**Authors:** Lisan M. Hidding, Teatske M. Altenburg, Evi van Ekris, Mai J. M. Chinapaw

**Affiliations:** Department of Public and Occupational Health, Amsterdam Public Health Institute, VU University Medical Center, 1081 BT Amsterdam, The Netherlands; t.altenburg@vumc.nl (T.M.A.); e.vanekris@vumc.nl (E.v.E.); m.chinapaw@vumc.nl (M.J.M.C.)

**Keywords:** sedentary activities, concept mapping, child, parent, determinants

## Abstract

Todays children spend a large amount of their time sedentary. There is limited evidence on the determinants of sedentary behavior in children, and qualitative studies are especially lacking. Therefore, this study aimed to explore determinants of children’s sedentary behavior from the child- and parent perspective. Qualitative data were collected during concept mapping sessions with four groups of 11–13 years old children (*n* = 38) and two online sessions with parents (*n* = 21). Children and parents generated sedentary behavior motives, sorted related motives, and rated their importance in influencing children’s sedentary time. Next, multidimensional scaling and hierarchical cluster analysis was performed to create clusters of motives resulting in a concept map. Finally, the researchers named the clusters in the concept map. Concept maps of children yielded eight to ten perceived determinants, and concept maps of parents six to seven. Children and parents identified six similar potential determinants, and both rated as important: Sitting because… “it is the norm (I have to)”, and “I can work/play better that way”. In addition, children rated “there is nobody to play with” as an important potential determinant for engaging in sedentary behavior. The most important child- and parent perceived determinants were related to the social/cultural and physical environment, indicating that these are promising targets for future interventions.

## 1. Introduction

Worldwide, the majority of children spend more than two hours per day watching TV [1,2], and laptop and phone use in children is increasing substantially [3]. Besides screen activities children engage in many other sedentary behaviors, e.g., sitting in the classroom and doing homework [4,5]. Studies in Europe and the United States, show that children spend approximately 8 h per day sedentary [6,7].

Recently, interest in the health consequences of excessive sedentary behavior is increasing. Systematic reviews only including longitudinal studies concluded no convincing evidence for a prospective relationship between childhood sedentary behavior and various health outcomes [8,9]. However, sedentary behavior tracks from early childhood to later in life [10,11], and for adults evidence for an adverse relationship between sedentary behavior and morbidity and mortality is emerging [12,13]. Therefore, excessive sedentary behavior among children remains a public health concern and developing interventions to reduce sedentary behavior seems appropriate. Unfortunately, existing interventions targeting reductions in children’s sedentary time have disappointing effects, possibly explained by a lack of knowledge on the most important determinants for engaging in sedentary behavior [14].

Previous reviews found that the most commonly examined determinants can be grouped in the following domains: (1) demographic and biological; (2) psychological, cognitive and emotional; (3) behavioral; (4) social and cultural; and (5) physical environmental [15,16,17,18]. Recent reviews found insufficient evidence for determinants of sedentary behavior in children in these five domains, as few prospective studies were available, and the majority of the determinants were examined only once. Furthermore, most studies focused on screen time such as watching television or playing videogames as an indicator of sedentary behavior [17,19,20]. Yet screen time takes up only a small part of the total time spent sedentary [5,21,22,23]. Finally, Brug and Chinapaw [24] state that many studies exploring determinants were not specifically designed for this question, and the determinants identified in the different reviews mainly concern characteristics of sedentary children instead of motivational and contextual reasons for engaging in sedentary behavior, which is essential for the design of effective interventions.

Another major gap in the current evidence on determinants is the perspective of children on why they engage in sedentary behavior. As children are the experts of their own behavior [25], their perspectives could bring new insights into relevant determinants of sedentary behavior. Furthermore, as parents play a significant role in facilitating and regulating their children’s behavior [26], their view on potential determinants of their children’s sedentary behavior is of additional relevance. Previous studies found, for example, that parents’ screen time, and parents’ rules concerning screen time and playing outside, were associated with children’s sedentary time [27,28,29]. Therefore, the aim of this study was to explore child- and parent-perceived determinants of children’s sedentary behavior, using concept mapping with 11 to 13 year old children and their parents. Concept mapping is a helpful participatory method to acquire knowledge about children’s and parents’ perspectives, by allowing them to provide their unique contribution to research concerning their own behavior [30,31].

## 2. Materials and Methods

### 2.1. Participants

Children and one group of parents were recruited via different primary schools in The Netherlands. Between April and October 2015 primary schools were selected through purposive sampling, contacted through information letters and follow-up telephone calls, aiming to include both rural and urban primary schools comprising children with different socio-economic backgrounds. After approaching 26 primary schools, the first school was included (response rate 4%). Thereafter, through purposive sampling based on socio-economic background and rural/urban area three other schools were contacted and included (response rate 100%). Thirty-eight children (response rate 40%) and seven parents were willing to participate (response rate 12%). Due to late inclusion of the fourth primary school, parents of the children at this school were not able to participate in the online concept mapping sessions. Therefore, a second group of parents (*n* = 14) were recruited through convenience sampling in January 2017.

When a school agreed to cooperate, information letters were sent to the children in the 8th grade, aged between 11–13 years old, and their parents. Both parents from non-participating and participating children were allowed to take part, as we were interested in the ideas of parents in general and not specifically the parents of participating children. Parents from the convenience sample received an information letter via email. As a reward for participation children received a small present, parents were offered an update on the results of the study, and the schools were offered a presentation about the results of the study.

The VU University Medical Ethical Committee concluded that the protocol does not fall within the scope of the Medical Research Involving Human Subjects Act. No identifying participant information was collected for the purpose of this study, and written informed consent was signed by one parent and the participating child, or the participating parent.

### 2.2. Procedures

Concept mapping is a research method in which group data are collected qualitatively in a structured and inductive way, and are analyzed quantitatively. The concept mapping procedure consists of several steps: generation of participants’ statements towards a seeding statement, grouping all statements and rating of their importance, summarizing these data into computer generated concept maps, and interpretation of these concept maps [30,31]. In this way, structure and objectivity to qualitative data are provided [30,31]. Generally, concept mapping studies include an average of 10–20 participants per study [31].

Concept mapping sessions and analyzes were performed between May 2015 and February 2017. At the start of the concept mapping sessions, gender and age were inquired from children, and from parents their age, gender, family structure, amount of siblings, and highest educational level, i.e., pre-vocational-, vocational-, or higher education. The socio-economic status (SES) of the primary schools was assessed based on zip codes of the schools and the status-scores document from the Dutch Social and Cultural Planning Agency. This SES score was divided in tertiles, i.e., low SES, medium SES, or high SES. The whole concept mapping procedure is described in detail below.

#### 2.2.1. Concept Mapping Sessions—Children

The concept mapping sessions with the children took place at their school during school hours, lasting approximately 2 h each. Four groups of 8–10 children, attending different primary schools, participated in these sessions that were facilitated by two trained researchers. Because of children’s limited attention span the whole procedure was divided over two sessions.

The first session comprised a warming up exercise, an individual brainstorm, and a group brainstorm. The purpose of the warming-up exercise was to broaden children’s understanding about sedentary behaviors, i.e., children wrote down their daily activities and divided them in active and inactive activities. Thereafter, the seeding statement was presented in two ways:

Can you think of different reasons why you choose to do activities in which you have a seated position?

“For some things I do, I do them in a seated position, I do these things because”

Subsequently, the individual brainstorm started, in which the participants were stimulated to finish the sentence with as many ideas as possible, and to write down all ideas. In doing this, they were stimulated to think about their own sedentary behavior, as well as the sedentary behaviors of 11–13-year old children in general. Thereafter, participants shared one by one all of their statements in the group brainstorm. This resulted in a list of unique statements.

In the second session, each child individually grouped the statements, printed on separate cards, into piles of related statements. A minimum of three and a maximum of ten piles were required due to settings of the software program, with at least two statements per pile, and a miscellaneous pile was prohibited. Subsequently, children named the different piles. Additionally, children individually rated the importance of the statements in influencing their sedentary time using a 5-point Likert-scale, ranging from very unimportant to very important.

#### 2.2.2. Concept Mapping Sessions—Parents

Concept mapping sessions with the parents were performed online, making it easier for the parents to participate. A comparable procedure as with the children was followed, i.e., a comparable warming-up exercise and the individual brainstorm towards the seeding statement were performed by e-mail, thereby omitting the group brainstorm. The sorting, naming and rating tasks were performed by means of the online program Ariadne [32]. The seeding statement was presented in two ways:

Can you think of different reasons why your child chooses to do activities in which he/she has a seated position?

“For some things my child does, he/she does them in a seated position, he/she does these things because…”

### 2.3. Statistical Analysis

Separate analyses were carried out for each school and two groups of parents. The data were analyzed by multidimensional scaling and hierarchical cluster analyses using the software program “Ariadne” [32], specifically designed for concept mapping. These analyses showed every statement as a point on a figure, with statements sorted more often together appearing close to each other, and statements never/rarely sorted together widely separated. By default eight clusters were made, combining statements close to each other. Subsequently, three independent researchers (Lisan M. Hidding, Teatske M. Altenburg, and Evi van Ekris) explored whether more or less clusters would represent participant’s ideas more adequate, i.e., each cluster comprising statements related to a similar concept. Based on the importance rating of the individual statements, average ratings for each statement and each cluster were calculated.

### 2.4. Interpretation of the Maps

Two researchers (LH and TA) interpreted the maps, by discussing similarities in the meaning of statements in each cluster, and subsequently, by determining the concept represented by each cluster. Next, researchers (LH and TA) named the clusters, far as possible derived from the names given by the participants in the individual sorting task. As statistical significance not always results in the best representation of the qualitative data, the researchers critically reflected on the computer-generated clusters. According to the researchers, some statements were not optimally represented in the computer-generated cluster maps. To improve the logic of the concept map, these statements were moved to nearby clusters or newly created clusters, the latter where possible based on clusters created by the participants in the individual sorting task. Original and final cluster compositions are shown in the supplemental material (Appendix A1 and Appendix A2, respectively). Finally, the maps were discussed, listing similarities and differences between the maps of the different primary schools and between the maps of the children and the parents, and an explanation for the findings was searched. A third researcher (Mai. J. M Chinapaw or Evi van Ekris) was consulted in case of disagreement. In the results section the final clusters are presented. The final clusters were interpreted as potential determinants as perceived by children and parents. Whether these potential determinants are actual determinants cannot be concluded based on this concept mapping study.

## 3. Results

### 3.1. Participants

The participating children (*n* = 38; 55% girls) were on average 11.6 ± 0.6 years old. Parents (*n* = 21; 71% female) were on average 42.1 ± 4.3 years old. One parent was part of a single-parent family and 20 were part of traditional dual-parent families, three comprising one child and 18 more than one child. Furthermore, two parents followed pre-vocational-, three parents vocational-, and 16 parents higher education.

### 3.2. School Characteristics

The four participating schools had a varying degree of urbanization: located in a small size village, a medium size village, a large size village and a large city. Two of the schools received a medium and two a low SES indication. Concept mapping groups varied from 8 to 10 children generating 31 to 51 statements.

### 3.3. Children’s Clusters

**Figure 1:**
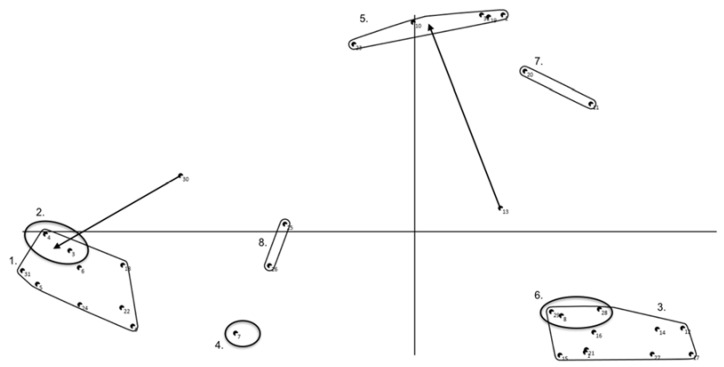
Concept map children (school 2). Note that points, i.e., statements, sorted more often together appear closer to each other, and statements never/rarely sorted together appear widely separated.; Arrows indicate a statement is reallocated by researchers, circles indicate a new cluster is created by researchers as a result of reallocation of statements.; Cluster 1: I sit because I can work/play better that way; Cluster 2: I sit because it is the norm/I sit because I have to; Cluster 3: I sit because I’m tired, I want to relax, I want to rest; Cluster 4: I sit because it is a habit; Cluster 5: I sit because there is nobody to play with; Cluster 6: I sit because seated activities are fun; Cluster 7: I sit because of the weather; Cluster 8: I sit because being active takes a lot of effort.

Recurrent clusters based on children’s concept maps (see Figure 1 for an example, see Appendix A3 for other maps), i.e., present in the concept maps of at least three schools, were: I sit because… (1) “it is the norm/I have to”; (2) “seated activities are fun”; (3) “I’m tired, I want to relax, I want to rest”; (4) “there is nobody to play (actively) with”; (5) “there is nothing to do”; (6) “I can work/play better that way”; and (7) “of the weather”. Additional clusters in the concept maps of one or two schools were: I sit because… (8) “I’m not in the mood to do anything”; (9) “of my health”; (10) “it feels better”; (11) “being active takes a lot of effort”; (12) “it is a habit”; (13) “this posture suits the activity better”; (14) “the physical environment suitable for physical activities is too far away”; (15) “the physical environment is not suitable”; and (16) “I want to make contact with my friends”.

Children of all primary schools rated the cluster “I sit because it is the norm/I have to” as most or second most important motive for engaging in sedentary behavior. Additionally, “I sit because I can work/play better that way”, “I sit because there is nobody to play with”, and “I sit because I want to make contact with my friends” were rated as most or second most important motives. Table 1 presents the child-identified clusters and average importance ratings for each school.

### 3.4. Parents’ Clusters

**Figure 2:**
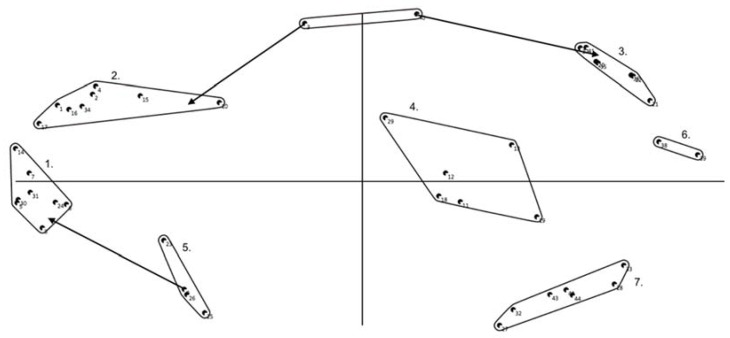
Concept map parents (convenience sample). Note that points, i.e., statements, sorted more often together appear closer to each other, and statements never/rarely sorted together appear widely separated.; Arrows indicate a statement is reallocated by researchers, circles indicate a new cluster is created by researchers as a result of reallocation of statements. Cluster 1: My child sits because it is the norm; Cluster 2: My child sits because he/she can work/play better that way; Cluster 3: My child sits because seated activities are fun; Cluster 4: My child sits because he/she is tired, wants to relax, wants to rest; Cluster 5: My child sits because others do so, and it is a habit; Cluster 6: My child sits because it is in his/her nature; Cluster 7: My child sits because there is nothing (active) to do.

The parents generated 24 (purposive sample), and 44 (convenience sample) statements, resulting in six and seven clusters, respectively (see Figure 2 for an example, see Appendix A3 for the other map). Recurrent clusters across both maps were: My child sits because… (1) “he/she can work/play better that way”; (2) “it is the norm”; (3) “there is nothing (active) to do”; (4) “he/she is tired, wants to relax, wants to rest”; (5) “seated activities are fun”; and (6) “it is a habit (and others do so)”. The cluster “My child sits because it is in his/her nature” was only found on one of the maps.

Both groups of parents rated “My child sits because he/she can work/play better that way” as second most important potential determinant. The most important potential determinant differed per group, i.e., “My child sits because he/she is tired, wants to relax, wants to rest”, and “My child sits because it is the norm”. Table 2 presents the parent-identified clusters with their average importance rating.

## 4. Discussion

To the best of our knowledge this is the first study exploring child- and parent-perceived determinants of children’s sedentary behavior, by performing concept mapping. Children and parents indicated several matching potential determinants, of which “I sit because I can work/play better that way” as one of the most important perceived determinants according to both children and parents. In addition, children indicated “I sit because it is the norm/I sit because I have to” and “I sit because there is nobody to play with” as considerable important determinants of their sedentary behavior.

The potential determinants found in our study fit within the frequently used domains to group determinants [15,16,17,18]. The majority of child- and parent-identified determinants belong to the psychological, cognitive and emotional domain, e.g., “I sit because I’m not in the mood to do anything”, and “I sit because seated activities are fun”. However, the perceived determinants that children and parents rated as most important, i.e., “I sit because of the norm/I sit because I have to” (children, one group of parents), “I sit because I can work/play better that way” (children, all parents), and “I sit because there is nobody to play with (children), in particular belong to the social and cultural domain and the physical environmental domain”. Interestingly, determinants belonging to the demographic and biological domain are extensively discussed in previous studies [17,19], yet they received far less attention in the present study, only one group of parents indicated such a determinant, i.e., “My child sits because it is in his/her nature”. In addition, determinants belonging to the behavioral domain received no attention.

The potential determinants “Sitting because it is the norm/Sitting because I have to” and “Sitting because I can work/play better that way” mentioned by both children and parents both include motives related to the current societal norm of a sedentary lifestyle. Interestingly, one group of parents rated “Sitting because it is the norm” as unimportant, while children and the second group of parents rated it as one of the most important motives for engaging in sedentary behavior. By establishing rules, and commanding children to sit during activities, some parents might unconsciously stimulate their children’s sedentary behavior [27,33]. Owen et al. [34] already found the norm to be a potential determinant in adults, as nowadays it is the norm to sit during many activities, including meetings, classes, and while relaxing at home. Similarly, many of the statements in the “Sitting because I can work/play better that way” clusters relate to the school or home environment, e.g., it is easier to work/play in a sedentary posture as their school environment is decorated for sedentary activities. Lanningham-Foster et al. [35] confirm that the school environment influences children’s sedentary behavior, as children were significantly more physically active when a classroom was made more suitable for active behaviors, e.g., availability of mobile white boards for active learning lessons.

One group of children and parents identified “Sitting because it is a habit” as a potential determinant. This finding is in line with studies in adolescents: de Bruijn et al. [36] found that habit strength was the strongest correlate of television viewing and Chinapaw et al. [37] found that changes in habit strength were significantly related to changes in screen time. In young adults Conroy et al. [38] found that habit strength had a strong bivariate correlation with objectively assessed sedentary behavior. Thus far, no studies examined the relation of habit strength with total sedentary time in children. The mechanism behind habit as a behavioral determinant is previously described in relation to exercise behavior. When behavior is a habit it is automatically activated by environmental stimulus or cues, instead of consciously deciding to engage in the behavior [39]. Due to the current societal norm of a sedentary lifestyle, children learn to be sedentary from an early age. Consequently, sedentary behavior has become a habit, e.g., ‘My child sits because others do so, and it is a habit’ indicated by one group of parents. This cluster additionally indicates that children also learn to sit by imitating others.

Children rated “I sit because there is nobody to play (actively) with” as an important potential determinant of their sedentary behavior, related statements were found in the cluster “Siting because there is nothing (active) to do” which was also indicated by their parents. This might imply that children naturally prefer physically active play but tend to sit when they have nothing to do or nobody to play with. This is in line with the participatory study of Caro et al. [40], where children preferred to be physically active in the school playground and were sedentary when they were bored. The children in this study perceived both social and physical environmental factors of the playground to influence fun of physically active play.

The children also indicated weather conditions as potential determinants, e.g., coldness, rain, or too hot temperatures within the clusters “I sit because of the weather” and “I sit because the physical environment is not suitable”. Additionally, they indicated statements related to safety and distance. Yilderim et al. [41], found a significant positive association between rainfall and sedentary time, confirming that the weather may indeed be a determinant of sedentary behavior. Additionally, a review found that parents may restrict children in playing outside when they have concerns about the safety of the neighborhood [42]. In our study only the children mentioned statements regarding safety of the neighborhood, indicating that it is not just a concern of their parents, however, children might have adopted their parent’s ideas.

Both children and parents identified enjoying sedentary activities as a potential determinant of sedentary behavior. Previous studies emphasized the importance of fun to children’s behavior as well. Norman et al. [43] found a positive association between the enjoyment of sedentary activities and high amounts of sedentary time. Moreover, a review found evidence for a negative association between preference of sedentary activities and time spent on physical activity [17].

Children mentioned statements related to feeling not so well and feeling bored, indicating children’s mood is another potential determinant of their sedentary behavior. This finding is in line with previous research indicating that lower psychological wellbeing was associated with higher TV/Video viewing and computer use during adolescence [44] and higher TV viewing later in life [45]. Rideout et al. [3] found that children who spent more time using media, e.g., mobile/online media and television, were more likely to feel unhappy, sad or bored.

The influence of new media on children’s behavior is highlighted in the cluster “I sit because I want to make contact with my friends”. Underlying statements show the influence of smartphones, social media, and online games. Children use these platforms and devices to keep in touch with their friends and family.

### 4.1. Strengths and Limitations

An important strength of this study is the focus on both child- and parent perspectives, giving not only new insights in potential determinants but also their relevance/importance in influencing sedentary time. Yet the use of the concept mapping method further strengthens this study by structuring qualitative data in a quantitative way and providing the opportunity to visualize the composite ideas of a group in one map, including participants from different backgrounds. In addition, the method consists of several educational tasks, e.g., sharing ideas in a group context, and sorting related motives into piles. Furthermore, concept mapping gives all participants the opportunity to share their individual ideas, as children share their ideas one by one during the group brainstorm, and parents provided their individual ideas by e-mail. Despite that a few children needed more guidance during the sorting task concept mapping proved a feasible and valuable method for this age group. The online concept mapping sessions with the parents may have reduced social desirable answers.

A limitation of the study is the limited age range of the children (11–13 years old). A strength is that a diverse group of children participated, i.e., with low and medium SES, living in urban and rural areas. In addition, similar clusters were identified across schools, and at the last school no new topics emerged in the underlying statements (i.e., indicating saturation), thereby strengthening the evidence for the representativeness of our findings for 11–13 years old in The Netherlands. Similarly, in the second group of parents no new topics emerged. The participating parents, who were not necessarily the parents of the participating children, were mainly highly educated, which may have limited the representativeness of parent-identified determinants. The low recruitment rate among parents is an additional limitation of our study, which may have resulted in selection bias. Lastly, a few children had difficulties with the sorting task, resulting in more statements being rearranged by researchers in the final concept maps. As the rearranged statements were included in the clusters emerging from the individual sorting task of the other children, the final concept maps are still representative for children’s perspectives.

### 4.2. Future Recommendations

Although the evidence for the adverse health effects of excessive sedentary behavior in youth is unconvincing, there is little potential harm of implementing sedentary time limiting interventions [8]. Current interventions aiming to reduce sedentary behavior mainly focused on determinants in the psychological, cognitive, emotional domain and behavioral domain. Strategies include for example enhancing children’s knowledge and awareness of their sedentary time, goal setting, and limiting TV time e.g., by using TV budgets [14]. Our study suggests that these may not be the most important determinants for children. The children and most parents in our study rated potential determinants belonging to the social/cultural and the physical environmental domain as most important, namely “I sit because of the norm/I sit because I have to”, and “I sit because I can work/play better that way”. These child- and parent-perceived determinants call for very different intervention strategies targeting the social or physical environment, giving them more freedom being non-sedentary e.g., by allowing children to be active during lessons, homework or screen time or providing gymnastic balls, standing and exercise desks. Additionally, future interventions might benefit from focusing on both the school and home setting, as children indicate being limited by physical activity restricting rules and environments in both settings. Moreover, involving both children and parents in the development of future interventions seems important, as their perceptions were not identical. Lastly, interventions targeting sedentary behavior reductions may be incorporated in interventions promoting moderate-to-vigorous physical activity, for which ample evidence exists of its beneficial health effects in youth. Although, whether the identified factors are indeed determinants of sedentary behavior should first be confirmed in prospective observational and intervention studies. In addition to perceived determinants of overall sedentary behavior, future studies should focus on child- and parent-perceived determinants of specific sedentary behaviors such as screen time and sitting at school, as it is likely that determinants of these behaviors may deviate from that of overall sedentary behavior.

## 5. Conclusions

This study adds important new insights into potential determinants of children’s sedentary behavior. Interestingly, parents’ and children’s perspectives differed with regard to both type and importance of potential determinants. This highlights the importance of involving children in research concerning their own behavior. The most important child-perceived determinants of sedentary behavior were: “I sit because of the norm/I sit because I have to”, “I sit because I can work/play better that way” and “I sit because there is nobody to play with”. Future observational and intervention studies are needed to confirm the importance of these potential determinants.

## Figures and Tables

**Table 1:** Children’s clusters and average importance ratings.

	Clusters	Importance ^a^
**School 1**		
	1. I sit because I can work/play better that way	4.4
	2. I sit because it is the norm/I sit because I have to	3.9
	3. I sit because it feels better	3.5
	4. I sit because seated activities are fun	3.4
	5. I sit because I’m tired, I want to relax, I want to rest	3.3
	6. I sit because of my health	3.1
	7. I sit because there is nobody to play with	3.0
	8. I sit because there is nothing to do	2.7
	9. I sit because I’m not in the mood to do anything	2.6
	10. I sit because of the weather	2.4
**School 2**		
	1. I sit because I can work/play better that way	3.7
	2. I sit because it is the norm/I sit because I have to	3.6
	3. I sit because I’m tired, I want to relax, I want to rest	3.4
	4. I sit because it is a habit	3.3
	5. I sit because there is nobody to play with	3.3
	6. I sit because seated activities are fun	3.2
	7. I sit because of the weather	3.1
	8. I sit because being active takes a lot of effort	2.7
**School 3**		
	1. I sit because it is the norm/I sit because I have to	3.9
	2. I sit because there is nobody to play with	3.7
	3. I sit because seated activities are fun	3.5
	4. I sit because I’m tired, I want to relax, I want to rest	3.5
	5. I sit because I’m not in the mood to do anything	3.5
	6. I sit because there is nothing to do	3.4
	7. I sit because this posture suits the activity better	3.4
	8. I sit because of the weather	3.1
	9. I sit because the physical environment suitable for physical activities is too far away	3.0
**School 4**		
	1. I sit because it is the norm/I sit because I have to	3.6
	2. I sit because I want to make contact with my friends	3.4
	3. I sit because I can work/play better that way	3.4
	4. I sit because there is nobody to play (actively) with	3.2
	5. I sit because I’m tired, I want to relax, I want to rest	3.0
	6. I sit because the physical environment is not suitable	3.0
	7. I sit because seated activities are fun	2.9
	8. I sit because there is nothing to do	2.8

^a^ Rated on a 5-point Likert scale with higher scores indicating higher importance in influencing children’s sedentary time.

**Table 2:** Parents’ clusters and average importance ratings.

	Clusters	Importance ^a^
**Parents (purposive sample)**		
	1. My child sits because he/she it tired, wants to relax, wants to rest	4.2
	2. My child sits because he/she can work/play better that way	4.0
	3. My child sits because seated activities are fun	3.4
	4. My child sits because there is nothing to do	2.6
	5. My child sits because it is the norm	2.5
	6. My child sits because it is a habit	2.4
**Parents (convenience sample) ^b^**		
	1. My child sits because it is the norm	3.5
	2. My child sits because he/she can work/play better that way	3.5
	3. My child sits because seated activities are fun	3.3
	4. My child sits because he/she is tired, wants to relax, wants to rest	3.3
	5. My child sits because others do so, and it is a habit	2.9
	6. My child sits because it is in his/her nature	2.8
	7. My child sits because there is nothing (active) to do	2.5

^a^ Rated on a 5-point Likert scale with higher scores indicating higher importance in influencing children’s sedentary time; ^b^ Sorting task *n* = 14, rating task *n* = 13.

**Figure A1:**
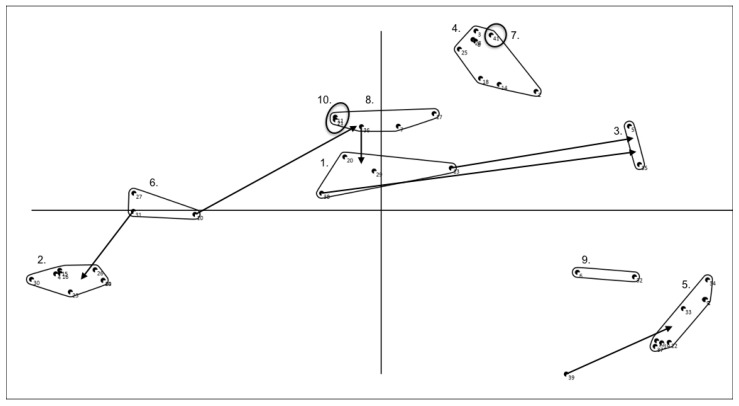
Concept map children (school 1). Note that points, i.e., statements, sorted more often together appear closer to each other, and statements never/rarely sorted together appear widely separated; Arrows indicate a statement is reallocated by researchers, circles indicate a new cluster is created by researchers as a result of reallocation of statements; Cluster 1: I sit because I can work/play better that way; Cluster 2: I sit because it is the norm/I sit because I have to; Cluster 3: I sit because it feels better; Cluster 4: I sit because seated activities are fun; Cluster 5: I sit because I’m tired, I want to relax, I want to rest; Cluster 6: I sit because of my health; Cluster 7: I sit because there is nobody to play with; Cluster 8: I sit because there is nothing to do; Cluster 9: I sit because I’m not in the mood to do anything; Cluster 10: I sit because of the weather.

**Figure A2:**
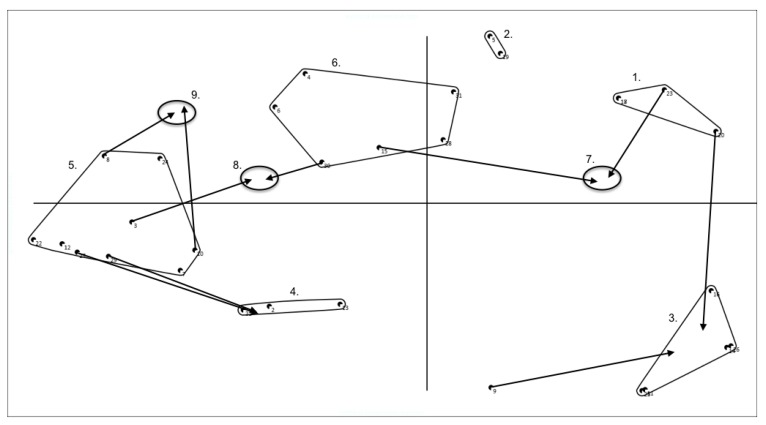
Concept map children (school 3). Note that points, i.e., statements, sorted more often together appear closer to each other, and statements never/rarely sorted together appear widely separated; Arrows indicate a statement is reallocated by researchers, circles indicate a new cluster is created by researchers as a result of reallocation of statements; Cluster 1: I sit because it is the norm/I sit because I have to; Cluster 2: I sit because there is nobody to play with; Cluster 3: I sit because seated activities are fun; Cluster 4: I sit because I’m tired, I want to relax, I want to rest; Cluster 5: I sit because I’m not in the mood to do anything; Cluster 6: I sit because there is nothing to do; Cluster 7: I sit because this posture suits the activity better; Cluster 8: I sit because of the weather; Cluster 9: I sit because the physical environment suitable for physical activities is too far away.

**Figure A3:**
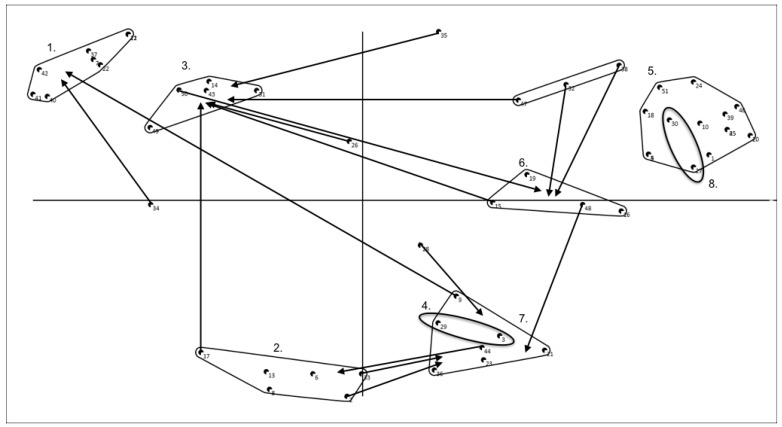
Concept map children (school 4). Note that points, i.e., statements, sorted more often together appear closer to each other, and statements never/rarely sorted together appear widely separated; Arrows indicate a statement is reallocated by researchers, circles indicate a new cluster is created by researchers as a result of reallocation of statements; Cluster 1: I sit because it is the norm/I sit because I have to; Cluster 2: I sit because I want to make contact with my friends; Cluster 3: I sit because I can work/play better that way; Cluster 4: I sit because there is nobody to play (actively) with; Cluster 5: I sit because I’m tired, I want to relax, I want to rest; Cluster 6: I sit because the physical environment is not suitable; Cluster 7: I sit because seated activities are fun; Cluster 8: I sit because there is nothing to do.

**Figure A4:**
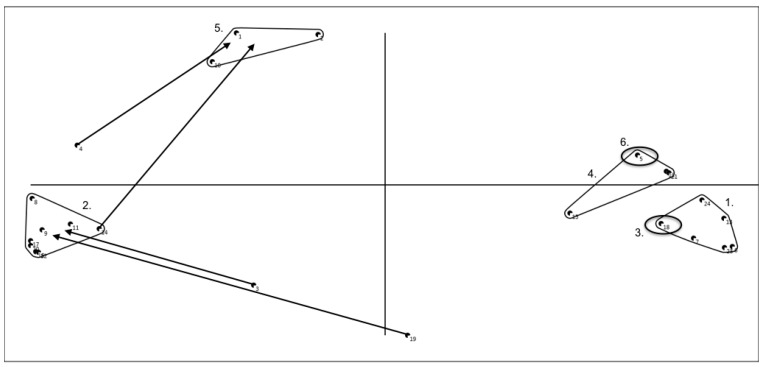
Concept map parents (purposive sample). Note that points, i.e., statements, sorted more often together appear closer to each other, and statements never/rarely sorted together appear widely separated; Arrows indicate a statement is reallocated by researchers, circles indicate a new cluster is created by researchers as a result of reallocation of statements; Cluster 1: My child sits because he/she it tired, wants to relax, wants to rest; Cluster 2: My child sits because he/she can work/play better that way; Cluster 3: My child sits because seated activities are fun; Cluster 4: My child sits because there is nothing to do; Cluster 5: My child sits because it is the norm; Cluster 6: My child sits because it is a habit.

## Supplementary Materials

Supplementary File 1

## Appendix A

### Appendix A.1. Original Cluster Compositions and Average Rating per Statement

**Table A1:** Clusters, statements and importance ratings of children (school 1).

	Clusters and Statements	Importance ^a^
1.	**I sit because it is the norm/I sit because I have to**	**3.86**
	4. I sit because what I’m doing has to be done seated	4.40
	15. I sit because it is safer to stay seated	4.30
	16. I sit because school, parents or grandparents say so	4.20
	23. I sit because otherwise the teacher gets mad	4.20
	24. I sit because I don’t want to draw attention to myself	3.60
	26. I sit because it is not logical to stand (weird)	3.30
	30. I sit because I’m not supposed to stand	3.80
	40. I sit because I feel people stare at me when I stand	3.10
2.	**I sit because I can work better that way**	**3.78**
	13. I sit because it is hard to stay standing up	3.20
	20. I sit because I can concentrate better this way	4.50
	29. I sit because it is easier to sit when I’m working	4.30
	38. I sit because I get nervous when I stand for too long	3.10
3.	**I sit because it feels better**	**3.75**
	5. I sit because it is cozy	3.80
	35. I sit because it is more comfortable	3.70
4.	**I sit because of a specific reason**	**3.40**
	10. I sit because I have to wait	3.20
	27. I sit because it is better for my digestion	3.10
	31. I sit because some activities are designed that way, gaming for example	3.90
5.	**I sit because seated activities are fun**	**3.34**
	2. I sit because I feel like it	2.90
	3. I sit because I like it	3.10
	8. I sit because the most fun things are seated activities	3.80
	14. I sit because it is nicer to sit	3.50
	18. I sit because there’s something good on TV	3.30
	25. I sit because it is more pleasant to sit	3.60
	28. I sit because sometimes seated activities are more fun	3.50
	41. When I’m alone I prefer to choose seated activities	3.00
6.	**I sit because I’m tired, I want to relax, I want to**	**3.30**
	1. I sit because I’m tired	3.60
	9. I sit because I want to rest	4.10
	12. I sit because I’m too lazy to stand	2.20
	19. I sit because it is useless to stand (costs energy)	3.30
	22. I sit because my legs are tired	2.90
	33. I sit to give my legs a rest	3.00
	34. I sit because I want to relax	4.00
	37. I sit because standing is tiring	3.30
7.	**I sit because there is nothing to do**	**2.80**
	7. I sit because I have nothing to do	2.40
	11. I sit because it is raining outside	2.70
	17. I sit because there is nothing to do	2.40
	21. I sit because it is too cold outside	2.10
	36. I sit because things go better that way, reading for example	4.40
8.	**I sit because I’m not in the mood to do anything**	**2.60**
	6. I sit because I’m bored	2.40
	32. I sit because I don’t feel like standing	2.80

^a^ Rated on a 5-point Likert scale with higher scores indicating higher importance. Remaining numbers which were not included in one of the clusters: 39. Because when I feel calm I sit.

**Table A2:** Clusters, statements and importance ratings of children (school 2).

	Clusters and Statements	Importance ^a^
1.	**I sit because I can work better that way/I sit because it is the norm/I sit because I have to**	**3.74**
	3. I sit because I have to at school	4.11
	4. I sit because there is no alternative	3.89
	5. I sit because I can concentrate better this way	4.89
	6. I sit because some activities can only be done seated	3.44
	9. I sit because it is more convenient	3.67
	18. I sit because I have a lot of homework, which I have to do on the computer	3.22
	22. I sit because it is easier	2.67
	24. I sit because I can think better that way	3.67
	31. I sit because writing while standing up is hard	4.11
2.	**I sit because there is nobody to play with**	**3.42**
	2. I sit because there are no other children to play with (in the neighborhood)	3.56
	10. I sit because my parents don’t always have the time to play with me	2.56
	19. I sit because it’s no fun playing outside on your own	4.00
	23. I sit because classmates live far away, and contact via the phone is easier	3.11
	32. I sit because you need two people to play outside, and it’s more fun with two	3.89
3.	**I sit because I’m tired, I want to relax, I want to rest/I sit because seated activities are fun**	**3.30**
	1. I sit because I want to rest	3.89
	8. I sit because it is fun	2.89
	12. I sit because it is comfortable to sit on the couch under a blanket	4.00
	14. I sit because standing for too long makes me tired	2.89
	15. I sit because I become calm	3.56
	16. I sit because sometimes I don’t feel like standing	3.33
	17. I sit because I’m tired	3.78
	21. I sit because it doesn’t cost any energy	2.78
	27. I sit because sitting is calmer	2.67
	28. I sit because I don’t want to miss my favorite TV-program	3.78
	29. I sit because sometimes it is more fun	2.78
4.	**I sit because of the weather**	**3.11**
	11. I sit because it is dark outside	2.56
	20. I sit because the weather is bad (cold, rain)	3.67
5.	**I sit because being active takes a lot of effort**	**2.67**
	25. I sit because I have to change my clothes if I want to play outside	2.44
	26. I sit because going outside takes a lot of effort (living on a farm)	2.89

^a^ Rated on a 5-point Likert scale with higher scores indicating higher importance. Remaining numbers which were not included in one of the clusters: 7. I sit because it is a habit; 13. I sit because you can use the computer when you are on your own; 30. I sit because my parents tell me to.

**Table A3:** Clusters, statements and importance ratings of children (school 3).

	Clusters and Statements	Importance ^a^
1.	**I sit because it is the norm/I sit because I have to**	**3.69**
	17. I sit because sometimes I have to sit at school	3.88
	18. I sit because my parents tell me to	3.88
	20. I sit because I’m asked to take part in a seated activity, gaming for example	3.38
	23. I sit because it’s easier talking with the family when you’re seated	3.63
2.	**I sit because there is nobody to play with**	**3.69**
	5. I sit because doing something on my own is stupid and I can make contact with the outside world via my phone	3.63
	19. I sit because I can’t arrange to meet people, so then I use the computer to make contact	3.75
3.	**I sit because I’m tired, I want to relax, I want to rest**	**3.46**
	2. I sit because I want to rest	3.38
	13. I sit because it is restful	3.38
	21. I sit because it is relaxed	3.63
4.	**I sit because seated activities are fun**	**3.40**
	11. I sit because I think it is fun	4.00
	14. I sit because sometimes it is more fun to do activities sitting down	3.38
	16. I sit because it is more logical	3.38
	25. I sit because it is fun	3.13
	26. I sit because a seated activity is more interesting	3.13
5.	**I sit because there is nothing to do**	**3.31**
	4. I sit because there are no nice people outside	3.38
	6. I sit because I have nothing to do	3.75
	15. I sit because standing is not convenient	3.13
	28. I sit because I have to wait a long time	3.50
	30. I sit because it is too hot	3.00
	31. I sit because I don’t know what to do	3.13
6.	**I sit because I’m not in the mood to do anything**	**3.31**
	1. I sit because I’m tired	3.63
	3. I sit because the weather is bad	3.25
	7. I sit because I don’t feel so well (not in the mood)	4.13
	8. I sit because I’m too far from somewhere I can take part in activities (the park)	3.25
	10. I sit because the seated activity is nearby	2.88
	12. I sit because I’m still sleepy	3.38
	22. I sit because I don’t feel like doing anything active	3.00
	24. I sit because I’m bored	3.13
	27. I sit because standing for too long makes me tired	3.25
	29. I sit because I already have to do a lot of standing	3.75

^a^ Rated on a 5-point Likert scale with higher scores indicating higher importance. Remaining numbers which were not included in one of the clusters: 9. I sit because I feel like it.

**Table A4:** Clusters, statements and importance ratings of children (school 4).

	Clusters and Statements	Importance ^a^
1.	**I sit because it is the norm/I sit because I have to**	**3.67**
	7. I sit because I’m not allowed to stand while learning in class	2.78
	11. I sit because some activities are impossible to do standing	3.89
	12. I sit because in some activities it is just the way it is done	3.78
	22. I sit because I have to eat	4.00
	37. I sit because it’s not possible to stand	3.78
	40. I sit because I have to pray	4.44
	41. I sit because in school I have to	3.44
	42. I sit because sometimes I’m not allowed to stand	3.22
2.	**I sit because I can work better that way**	**3.40**
	14. I sit because standing and running around can be distracting	2.56
	31. I sit because I have to concentrate while I’m working	4.00
	43. I sit because then you can work more accurately	3.44
	49. I sit because sometimes it is more polite	3.67
	50. I sit because some locations are too far to cycle, then I’ll go by car	3.33
3.	**I sit because seated activities are fun/I sit because I want to make contact with my friends**	**3.38**
	2. I sit because I like a particular game a lot	3.22
	6. I sit because a friend of mine lives far away, to keep in touch we talk to each other on the phone	4.00
	8. I sit because I play some games online with my friends	3.33
	13. I sit because I can keep in touch with friends online who live far away	3.78
	33. Children sit because they may be addicted to a computer game	2.56
4.	**I sit because of a specific reason**	**3.26**
	32. I sit because when you play hide and seek, people can see you when you stand	3.44
	38. I sit because sometimes being active is dangerous	2.89
	47. I sit because sometimes it is more pleasant for others when you sit	3.44
5.	**I sit because seated activities are fun/I sit because there is nobody to play (actively) with**	**3.13**
	3. I sit because there is nobody to play with	3.56
	9. I sit because when I have to learn I’ll do it on my computer	3.44
	21. I sit because I want to watch a program	3.00
	23. I sit because it is cozy to watch YouTube videos	3.44
	29. I sit because my friends want to play inside	2.78
	36. I sit because sometimes I want to laugh at funny videos	3.22
	44. I sit because I want to stay up to date with my social media	2.44
6.	**I sit because I’m tired, I want to relax, I want to rest**	**2.98**
	1. I sit because I’m tired	3.33
	4. I sit when something is bothering me	4.33
	5. I sit when I’m ill	4.00
	10. I sit because I prefer to sit	2.11
	18. I sit because you need less energy for seated activities	2.44
	20. I sit because I’m lazy	2.11
	24. I sit because it is more relaxing	3.67
	25. I sit because it is tiring to stand	1.89
	27. I sit because I’m bored	2.44
	30. I sit because when I’m bored it is an easy solution	3.11
	39. I sit because when you stand for too long you’ll get cramps	3.11
	45. I sit because standing activities are tiring	2.22
	46. I sit because I want to rest after a workout	3.78
	51. I sit because sometimes it is more pleasant	3.11
7.	**I sit because the physical environment is not suitable**	**2.75**
	15. I sit because I have to practice in order to be a game programmer when I’m older	2.89
	16. I sit because maybe the weather is bad outside	3.22
	19. I sit because in my neighborhood there are a lot bushes with thorns	2.33
	48. I sit because I love it	2.56

^a^ Rated on a 5-point Likert scale with higher scores indicating higher importance. Remaining numbers which were not included in one of the clusters: 17. I sit because I have to practice a lot to be able to play a game well; 26. I sit because I often learn for a good grade; 28. I sit because it is nice and funny; 34. I sit because with my parents I often do activities where I have to sit; 35. I sit because some things are more practical while seated.

**Table A5:** Clusters, statements and importance ratings of parents (purposive sample).

	Clusters and Statements	Importance ^a^
1.	**My child sits because he/she is tired, wants to relax, wants to rest**	**4.10**
	6. Sitting because it is a comfortable posture after some exercise	4.29
	7. Sitting because it is a relaxed posture	4.43
	13. Sitting because he/she wants to rest	4.14
	18. Sitting because he/she then enjoys him/herself	3.43
	23. Sitting because it is a comfortable posture	4.14
	24. Sitting because he/she can then rest	4.14
2.	**My child sits because he/she can work better that way**	**3.88**
	8. Sitting because the activity requires a sitting posture	4.14
	9. Sitting because it is more convenient	3.29
	11. Sitting because it suits the activity	4.29
	14. Sitting because he/she has to travel	3.57
	16. Sitting because he/she has to do homework	3.86
	17. Sitting because the situation demands it	4.00
	20. Sitting because he/she has schoolwork to do	4.14
	22. Sitting because he/she has to study	3.71
3.	**My child sits because there is nothing to do**	**2.62**
	5. Sitting by force of habit	2.43
	12. Sitting because there is nothing active to do	2.14
	15. Sitting to make contact with friends, chatting for example	3.43
	21. Sitting because he/she is bored	2.29
4.	**My child sits because it is the norm**	**2.14**
	1. Sitting because the interior is designed that way	2.00
	2. Sitting because other people in the social environment do it as well	1.71
	10. Sitting because it’s what has been taught	2.71

^a^ Rated on a 5-point Likert scale with higher scores indicating higher importance. Remaining numbers which were not included in one of the clusters: 3. Sitting because this is the most practical posture for performing the activity; 4. Sitting because he/she has been told to; 19. Sitting because he/she wants to work quietly.

**Table A6:** Clusters, statements and importance ratings of parents (convenience sample) ^a^.

	Clusters and Statements	Importance ^b^
1.	**My child sits because it is the norm**	**3.52**
	5. Sitting because he/she has to, e.g., at school or while eating/doing homework	4.15
	6. Sitting because he/she is not allowed to lie down	2.46
	7. Sitting because he/she has no other option than to sit or lie down during the activity	4.00
	8. Sitting because there are no desks at school or at home	3.46
	14. Sitting because the activity is designed for a sitting posture, e.g., the computer and TV are placed at sitting height	3.08
	24. Sitting because he/she has been taught to perform the activity in a sitting posture	2.92
	30. Sitting because he/she is expected to sit, e.g., at school, while doing homework or eating	4.23
	31. Sitting because he/she has been told to	3.85
2.	**My child sits because he/she can work/play better that way**	**3.47**
	1. Sitting because the activity requires a sitting posture	4.00
	2. Sitting because it is most appropriate for the activity	3.85
	4. Sitting because it is practical	3.15
	10. Sitting because he/she has to concentrate	3.15
	15. Sitting because he/she can perform the activity more easily that way, eating and doing a puzzle for example	3.15
	16. Sitting because it is a more natural posture for the activity	2.85
	17. Sitting because for some activities there is no other way, e.g., playing on a tablet, watching TV or travelling by car	4.23
	34. Sitting because it is safer to be seated during the activity, playing with a hamster for example	3.38
3.	**My child sits because he/she is tired, wants to rest/relax**	**3.33**
	20. Sitting because it is relaxing	3.62
	21. Sitting because he/she is tired	3.23
	22. Sitting because it is an important relaxation moment	3.62
	35. Sitting because he/she is in a relaxed mood	2.46
	36. Sitting because a seated activity can provide peace and quiet	3.54
	37. Sitting because he/she has to calm down physically	3.85
	41. Sitting because it is a relaxing activity	3.00
4.	**My child sits because seated activities are fun**	**3.32**
	11. Sitting because he/she likes to do seated activities	3.31
	12. Sitting because he/she feels like doing a seated activity	3.00
	13. Sitting because he/she enjoys doing a seated activity	3.54
	18. Sitting because he/she enjoys the activity	4.38
	19. Sitting because he/she likes the seated activity	3.31
	29. Sitting because he/she forgets the world around him/her, while watching TV for example	2.38
5.	**My child sits because its comfortable, and requires less energy**	**3.04**
	3. Sitting because a seated/lying posture is the most comfortable	3.31
	40. Sitting because he/she requires less energy for seated activities	2.77
6.	**My child sits because others do so, and it is a habit**	**2.94**
	9. Sitting because he/she lives in a sedentary society	3.15
	23. Sitting by force of habit	3.08
	25. Sitting because he/she copies sedentary behavior of the parents and others	3.00
	26. Sitting because others nearby are doing seated activities, e.g., watching TV	2.54
7.	**My child sits because it is in his/her nature**	**2.81**
	38. Sitting because he/she is naturally a quiet child	2.38
	39. Sitting because he/she wants to withdraw into him/herself	3.23
8.	**My child sits because there is nothing (active) to do**	**2.52**
	27. Sitting because there is not much to do (that encourages physical activity)	2.54
	28. Sitting because he/she is bored	2.31
	32. Sitting because I (as a parent) don’t have the time or don’t feel like doing something active with my child	2.23
	33. Sitting because he/she doesn’t feel like doing a more active activity, e.g., playing outside	3.00
	42. Sitting because some seated activities easily fill up the day, he/she doesn’t have to think about what he/she can do, e.g., watching TV	2.46
	43. Sitting because he/she can’t think of anything else to do	2.15
	44. Sitting because he/she has nobody to play with	2.92

^a^ Sorting task *n* = 14, rating task *n* = 13; ^b^ Rated on a 5-point Likert scale with higher scores indicating higher importance.

### Appendix A.2. Final Cluster Compositions and Average Rating per Statement

**Table A7:** Clusters, statements and importance ratings of children (school 1).

	Clusters and Statements	Importance ^a^
1.	**I sit because I can work better that way**	**4.40**
	20. I sit because I can concentrate better this way	4.50
	29. I sit because it is easier to sit when I’m working	4.30
	36. I sit because things go better that way, reading for example ^b^	4.40
2.	**I sit because it is the norm/I sit because I have to**	**3.86**
	4. I sit because what I’m doing has to be done seated	4.40
	15. I sit because it is safer to stay seated	4.30
	16. I sit because school, parents or grandparents say so	4.20
	23. I sit because otherwise the teacher gets mad	4.20
	24. I sit because I don’t want to draw attention to myself	3.60
	26. I sit because it is not logical to stand (weird)	3.30
	30. I sit because I’m not supposed to stand	3.80
	40. I sit because I feel people stare at me when I stand	3.10
	31. I sit because some activities are designed that way, gaming for example ^b^	3.90
3.	**I sit because it feels better**	**3.45**
	5. I sit because it is cozy	3.80
	35. I sit because it is more comfortable	3.70
	38. I sit because I get nervous when I stand for too long ^b^	3.10
	13. I sit because it is hard to stay standing up ^b^	3.20
4.	**I sit because seated activities are fun**	**3.39**
	2. I sit because I feel like it	2.90
	3. I sit because I like it	3.10
	8. I sit because the most fun things are seated activities	3.80
	14. I sit because it is nicer to sit	3.50
	18. I sit because there’s something good on TV	3.30
	25. I sit because it is more pleasant to sit	3.60
	28. I sit because sometimes seated activities are more fun	3.50
5.	**I sit because I’m tired, I want to relax, I want to rest**	**3.31**
	1. I sit because I’m tired	3.60
	9. I sit because I want to rest	4.10
	12. I sit because I’m too lazy to stand	2.20
	19. I sit because it is useless to stand (costs energy)	3.30
	22. I sit because my legs are tired	2.90
	33. I sit to give my legs a rest	3.00
	34. I sit because I want to relax	4.00
	37. I sit because standing is tiring	3.30
	39. Because when I feel calm I sit ^b^	3.40
6.	**I sit because of my health**	**3.10**
	27. I sit because it is better for my digestion	3.10
7.	**I sit because there is nobody to play with ^c^**	**3.00**
	41. When I’m alone I prefer to choose seated activities	3.00
8.	**I sit because there is nothing to do**	**2.67**
	7. I sit because I have nothing to do	2.40
	17. I sit because there is nothing to do	2.40
	10. I sit because I have to wait^b^	3.20
9.	**I sit because I’m not in the mood to do anything**	**2.60**
	6. I sit because I’m bored	2.40
	32. I sit because I don’t feel like standing	2.80
10.	**I sit because of the weather ^c^**	**2.40**
	11. I sit because it is raining outside	2.70
	21. I sit because it is too cold outside	2.10

^a^ Rated on a 5-point Likert scale with higher scores indicating higher importance; ^b^ Indicates a statement is reallocated by researchers; ^c^ Indicates a new cluster is created by researchers as a result of reallocation of statements.

**Table A8:** Clusters, statements and importance ratings of children (school 2).

	Clusters and Statements	Importance ^a^
1.	**I sit because I can work better that way**	**3.67**
	5. I sit because I can concentrate better this way	4.89
	6. I sit because some activities can only be done seated	3.44
	9. I sit because it is more convenient	3.67
	18. I sit because I have a lot of homework, which I have to do on the computer	3.22
	22. I sit because it is easier	2.67
	24. I sit because I can think better that way	3.67
	31. I sit because writing while standing up is hard	4.11
2.	**I sit because it is the norm/I sit because I have to ^c^**	**3.59**
	3. I sit because I have to at school	4.11
	4. I sit because there is no alternative	3.89
	30. I sit because my parents tell me to	2.78
3.	**I sit because I’m tired, I want to relax, I want to rest**	**3.36**
	1. I sit because I want to rest	3.89
	12. I sit because it is comfortable to sit on the couch under a blanket	4.00
	14. I sit because standing for too long makes me tired	2.89
	15. I sit because I become calm	3.56
	16. I sit because sometimes I don’t feel like standing	3.33
	17. I sit because I’m tired	3.78
	21. I sit because it doesn’t cost any energy	2.78
	27. I sit because sitting is calmer	2.67
4.	**I sit because it is a habit ^d^**	**3.33**
	7. I sit because it is a habit	3.33
5.	**I sit because there is nobody to play with**	**3.30**
	2. I sit because there are no other children to play with (in the neighborhood)	3.56
	10. I sit because my parents don’t always have the time to play with me	2.56
	19. I sit because it’s no fun playing outside on your own	4.00
	23. I sit because classmates live far away, and contact via the phone is easier	3.11
	32. I sit because you need two people to play outside, and it’s more fun with two	3.89
	13. I sit because you can use the computer when you are on your own ^b^	2.67
6.	**I sit because seated activities are fun ^c^**	**3.15**
	8. I sit because it is fun	2.89
	28. I sit because I don’t want to miss my favorite TV-program	3.78
	29. I sit because sometimes it is more fun	2.78
7.	**I sit because of the weather**	**3.12**
	11. I sit because it is dark outside	2.56
	20. I sit because the weather is bad (cold, rain)	3.67
8.	**I sit because being active takes a lot of effort**	**2.67**
	25. I sit because I have to change my clothes if I want to play outside	2.44
	26. I sit because going outside takes a lot of effort (living on a farm)	2.89

^a^ Rated on a 5-point Likert scale with higher scores indicating higher importance; ^b^ Indicates a statement is reallocated by researchers; ^c^ Indicates a new cluster is created by researchers as a result of reallocation of statements; ^d^ Indicates cluster based on a single statement, that was originally not included in a cluster.

**Table A9:** Clusters, statements and importance ratings of children (school 3).

	Clusters and statements	Importance ^a^
1.	**I sit because it is the norm/I sit because I have to**	**3.88**
	17. I sit because sometimes I have to sit at school	3.88
	18. I sit because my parents tell me to	3.88
2.	**I sit because there is nobody to play with**	**3.69**
	5. I sit because doing something on my own is stupid and I can make contact with the outside world via my phone	3.63
	19. I sit because I can’t arrange to meet people, so then I use the computer to make contact	3.75
3.	**I sit because seated activities are fun**	**3.49**
	11. I sit because I think it is fun	4.00
	14. I sit because sometimes it is more fun to do activities sitting down	3.38
	16. I sit because it is more logical	3.38
	25. I sit because it is fun	3.13
	26. I sit because a seated activity is more interesting	3.13
	20. I sit because I’m asked to take part in a seated activity, gaming for example ^b^	3.38
	9. I sit because I feel like it ^b^	4.00
4.	**I sit because I’m tired, I want to relax, I want to rest**	**3.48**
	2. I sit because I want to rest	3.38
	13. I sit because it is restful	3.38
	21. I sit because it is relaxed	3.63
	27. I sit because standing for too long makes me tired ^b^	3.25
	29. I sit because I already have to do a lot of standing ^b^	3.75
5.	**I sit because I’m not in the mood to do anything**	**3.45**
	1. I sit because I’m tired	3.63
	7. I sit because I don’t feel so well (not in the mood)	4.13
	12. I sit because I’m still sleepy	3.38
	22. I sit because I don’t feel like doing anything active	3.00
	24. I sit because I’m bored	3.13
6.	**I sit because there is nothing to do**	**3.44**
	4. I sit because there are no nice people outside	3.38
	6. I sit because I have nothing to do	3.75
	28. I sit because I have to wait a long time	3.50
	31. I sit because I don’t know what to do	3.13
7.	**I sit because this posture suits the activity better ^c^**	**3.38**
	15. I sit because standing is not convenient	3.13
	23. I sit because it’s easier talking with the family when you’re seated	3.63
8.	**I sit because of the weather ^c^**	**3.13**
	30. I sit because it is too hot	3.00
	3. I sit because the weather is bad	3.25
9.	**I sit because the physical environment suitable for physical activities is too far away ^c^**	**3.01**
	8. I sit because I’m too far from somewhere I can take part in activities (the park)	3.25
	10. I sit because the seated activity is nearby	2.88

^a^ Rated on a 5-point Likert scale with higher scores indicating higher importance; ^b^ Indicates a statement is reallocated by researchers; ^c^ Indicates a new cluster is created by researchers as a result of reallocation of statements.

**Table A10:** Clusters, statements and importance ratings of children (school 4).

	Clusters and Statements	Importance ^a^
1.	**I sit because it is the norm/I sit because I have to**	**3.61**
	7. I sit because I’m not allowed to stand while learning in class	2.78
	11. I sit because some activities are impossible to do standing	3.89
	12. I sit because in some activities it is just the way it is done	3.78
	22. I sit because I have to eat	4.00
	37. I sit because it’s not possible to stand	3.78
	40. I sit because I have to pray	4.44
	41. I sit because in school I have to	3.44
	42. I sit because sometimes I’m not allowed to stand	3.22
	9. I sit because when I have to learn I’ll do it on my computer ^b^	3.44
	34. I sit because with my parents I often do activities where I have to sit ^b^	3.33
2.	**I sit because I want to make contact with my friends**	**3.39**
	6. I sit because a friend of mine lives far away, to keep in touch we talk to each other on the phone	4.00
	8. I sit because I play some games online with my friends	3.33
	13. I sit because I can keep in touch with friends online who live far away	3.78
	44. I sit because I want to stay up to date with my social media ^b^	2.44
3.	**I sit because I can work better that way**	**3.37**
	14. I sit because standing and running around can be distracting	2.56
	31. I sit because I have to concentrate while I’m working	4.00
	43. I sit because then you can work more accurately	3.44
	49. I sit because sometimes it is more polite	3.67
	15. I sit because I have to practice in order to be a game programmer when I’m older ^b^	2.89
	47. I sit because sometimes it is more pleasant for others when you sit ^b^	3.44
	17. I sit because I have to practice a lot to be able to play a game well ^b^	2.78
	26. I sit because I often learn for a good grade ^b^	3.56
	35. I sit because some things are more practical while seated ^b^	4.00
4.	**I sit because there is nobody to play (actively) with ^c^**	**3.17**
	3. I sit because there is nobody to play with	3.56
	29. I sit because my friends want to play inside	2.78
5.	**I sit because I’m tired, I want to relax, I want to rest**	**3.01**
	1. I sit because I’m tired	3.33
	4. I sit when something is bothering me	4.33
	5. I sit when I’m ill	4.00
	10. I sit because I prefer to sit	2.11
	18. I sit because you need less energy for seated activities	2.44
	20. I sit because I’m lazy	2.11
	24. I sit because it is more relaxing	3.67
	25. I sit because it is tiring to stand	1.89
	39. I sit because when you stand for too long you’ll get cramps	3.11
	45. I sit because standing activities are tiring	2.22
	46. I sit because I want to rest after a workout	3.78
	51. I sit because sometimes it is more pleasant	3.11
6.	**I sit because the physical environment is not suitable**	**3.00**
	16. I sit because maybe the weather is bad outside	3.22
	19. I sit because in my neighborhood there are a lot bushes with thorns	2.33
	32. I sit because when you play hide and seek, people can see you when you stand ^b^	3.44
	38. I sit because sometimes being active is dangerous ^b^	2.89
	50. I sit because some locations are too far to cycle, then I’ll go by car ^b^	3.33
7.	**I sit because seated activities are fun**	**2.90**
	21. I sit because I want to watch a program	3.00
	23. I sit because it is cozy to watch YouTube videos	3.44
	36. I sit because sometimes I want to laugh at funny videos	3.22
	48. I sit because I love it ^b^	2.56
	28. I sit because it is nice and funny ^b^	2.33
	2. I sit because I like a particular game a lot ^b^	3.22
	33. Children sit because they may be addicted to a computer game ^b^	2.56
8.	**I sit because there is nothing to do ^c^**	**2.78**
	27. I sit because I’m bored	2.44
	30. I sit because when I’m bored it is an easy solution	3.11

^a^ Rated on a 5-point Likert scale with higher scores indicating higher importance; ^b^ Indicates a statement is reallocated by researchers; ^c^ Indicates a new cluster is created by researchers as a result of reallocation of statements.

**Table A11:** Clusters, statements and importance ratings of parents (purposive sample).

	Clusters and Statements	Importance ^a^
1.	**My child sits because he/she it tired, wants to relax, wants to rest**	**4.23**
	6. Sitting because it is a comfortable posture after some exercise	4.29
	7. Sitting because it is a relaxed posture	4.43
	13. Sitting because he/she wants to rest	4.14
	23. Sitting because it is a comfortable posture	4.14
	24. Sitting because he/she can then rest	4.14
2.	**My child sits because he/she can work better that way**	**3.97**
	8. Sitting because the activity requires a sitting posture	4.14
	9. Sitting because it is more convenient	3.29
	11. Sitting because it suits the activity	4.29
	16. Sitting because he/she has to do homework	3.86
	17. Sitting because the situation demands it	4.00
	20. Sitting because he/she has schoolwork to do	4.14
	22. Sitting because he/she has to study	3.71
	3. Sitting because this is the most practical posture for performing the activity ^b^	4.14
	19. Sitting because he/she wants to work quietly ^b^	4.14
3.	**My child sits because seated activities are fun ^c^**	**3.43**
	18. Sitting because he/she then enjoys him/herself	3.43
4.	**My child sits because there is nothing to do**	**2.62**
	12. Sitting because there is nothing active to do	2.14
	15. Sitting to make contact with friends, chatting for example	3.43
	21. Sitting because he/she is bored	2.29
5.	**My child sits because it is the norm**	**2.51**
	1. Sitting because the interior is designed that way	2.00
	2. Sitting because other people in the social environment do it as well	1.71
	10. Sitting because it’s what has been taught	2.71
	14. Sitting because he/she has to travel ^b^	3.57
	4. Sitting because he/she has been told to ^b^	2.57
6.	**My child sits because it is a habit ^c^**	**2.43**
	5. Sitting by force of habit	2.43

^a^ Rated on a 5-point Likert scale with higher scores indicating higher importance; ^b^ Indicates a statement is reallocated by researchers; ^c^ Indicates a new cluster is created by researchers as a result of reallocation of statements.

**Table A12:** Clusters, statements and importance ratings of parents (convenience sample) ^a^.

	Clusters and Statements	Importance ^b^
1.	**My child sits because it is the norm**	**3.47**
	5. Sitting because he/she has to, e.g., at school or while eating/doing homework	4.15
	6. Sitting because he/she is not allowed to lie down	2.46
	7. Sitting because he/she has no other option than to sit or lie down during the activity	4.00
	8. Sitting because there are no desks at school or at home	3.46
	14. Sitting because the activity is designed for a sitting posture, e.g., the computer and TV are placed at sitting height	3.08
	24. Sitting because he/she has been taught to perform the activity in a sitting posture	2.92
	30. Sitting because he/she is expected to sit, e.g., at school, while doing homework or eating	4.23
	31. Sitting because he/she has been told to	3.85
	9. Sitting because he/she lives in a sedentary society ^c^	3.15
2.	**My child sits because he/she can work/play better that way**	**3.45**
	1. Sitting because the activity requires a sitting posture	4.00
	2. Sitting because it is most appropriate for the activity	3.85
	4. Sitting because it is practical	3.15
	10. Sitting because he/she has to concentrate	3.15
	15. Sitting because he/she can perform the activity more easily that way, eating and doing a puzzle for example	3.15
	16. Sitting because it is a more natural posture for the activity	2.85
	17. Sitting because for some activities there is no other way, e.g., playing on a tablet, watching TV or travelling by car	4.23
	34. Sitting because it is safer to be seated during the activity, playing with a hamster for example	3.38
	3. Sitting because a seated/lying posture is the most comfortable ^c^	3.31
3.	**My child sits because seated activities are fun**	**3.32**
	11. Sitting because he/she likes to do seated activities	3.31
	12. Sitting because he/she feels like doing a seated activity	3.00
	13. Sitting because he/she enjoys doing a seated activity	3.54
	18. Sitting because he/she enjoys the activity	4.38
	19. Sitting because he/she likes the seated activity	3.31
	29. Sitting because he/she forgets the world around him/her, while watching TV for example	2.38
4.	**My child sits because he/she is tired, wants to rest/relax**	**3.26**
	20. Sitting because it is relaxing	3.62
	21. Sitting because he/she is tired	3.23
	22. Sitting because it is an important relaxation moment	3.62
	35. Sitting because he/she is in a relaxed mood	2.46
	36. Sitting because a seated activity can provide peace and quiet	3.54
	37. Sitting because he/she has to calm down physically	3.85
	41. Sitting because it is a relaxing activity	3.00
	40. Sitting because he/she requires less energy for seated activities ^c^	2.77
5.	**My child sits because others do so, and it is a habit**	**2.87**
	23. Sitting by force of habit	3.08
	25. Sitting because he/she copies sedentary behavior of the parents and others	3.00
	26. Sitting because others nearby are doing seated activities, e.g., watching TV	2.54
6.	**My child sits because it is in his/her nature**	**2.81**
	38. Sitting because he/she is naturally a quiet child	2.38
	39. Sitting because he/she wants to withdraw into him/herself	3.23
7.	**My child sits because there is nothing (active) to do**	**2.52**
	27. Sitting because there is not much to do (that encourages physical activity)	2.54
	28. Sitting because he/she is bored	2.31
	32. Sitting because I (as a parent) don’t have the time or don’t feel like doing something active with my child	2.23
	33. Sitting because he/she doesn’t feel like doing a more active activity, e.g., playing outside	3.00
	42. Sitting because some seated activities easily fill up the day, he/she doesn’t have to think about what he/she can do, e.g., watching TV	2.46
	43. Sitting because he/she can’t think of anything else to do	2.15
	44. Sitting because he/she has nobody to play with	2.92

^a^ Sorting task *n* = 14, rating task *n* = 13; ^b^ Rated on a 5-point Likert scale with higher scores indicating higher importance; ^c^ Indicates a statement is reallocated by researchers.
