# Supplementary material for: Why Do Children Engage in Sedentary Behavior? Child- and Parent-Perceived Determinants
Source: Int J Environ Res Public Health. 2017 Jun 22;14(7):671. doi: 10.3390/ijerph14070671 (PMC5551109; doi:10.3390/ijerph14070671)
Supplement: Supplementary File 1 [file ijerph-14-00671-s001.zip › ijerph-195872-S1.pdf]

Appendix S1 – Original cluster compositions and average rating per statement

Table 1. Clusters, statements and importance ratings of children (school 1).

	**Clusters** and statements	Importance[a]
1.	**I sit because it is the norm/I sit because I have to**	**3.86**
	4. I sit because what I'm doing has to be done seated	4.40
	15. I sit because it is safer to stay seated	4.30
	16. I sit because school, parents or grandparents say so	4.20
	23. I sit because otherwise the teacher gets mad	4.20
	24. I sit because I don't want to draw attention to myself	3.60
	26. I sit because it is not logical to stand (weird)	3.30
	30. I sit because I'm not supposed to stand	3.80
	40. I sit because I feel people stare at me when I stand	3.10
2.	**I sit because I can work better that way**	**3.78**
	13. I sit because it is hard to stay standing up	3.20
	20. I sit because I can concentrate better this way	4.50
	29. I sit because it is easier to sit when I'm working	4.30
	38. I sit because I get nervous when I stand for too long	3.10
3.	**I sit because it feels better**	**3.75**
	5. I sit because it is cozy	3.80
	35. I sit because it is more comfortable	3.70
4.	**I sit because of a specific reason**	**3.40**
	10. I sit because I have to wait	3.20
	27. I sit because it is better for my digestion	3.10
	31. I sit because some activities are designed that way, gaming for example	3.90
5.	**I sit because seated activities are fun**	**3.34**
	2. I sit because I feel like it	2.90
	3. I sit because I like it	3.10
	8. I sit because the most fun things are seated activities	3.80
	14. I sit because it is nicer to sit	3.50
	18. I sit because there's something good on TV	3.30
	25. I sit because it is more pleasant to sit	3.60
	28. I sit because sometimes seated activities are more fun	3.50
	41. When I'm alone I prefer to choose seated activities	3.00
6.	**I sit because I'm tired, I want to relax, I want to**	**3.30**
	1. I sit because I'm tired	3.60
	9. I sit because I want to rest	4.10
	12. I sit because I'm too lazy to stand	2.20
	19. I sit because it is useless to stand (costs energy)	3.30
	22. I sit because my legs are tired	2.90

	33. I sit to give my legs a rest	3.00
	34. I sit because I want to relax	4.00
	37. I sit because standing is tiring	3.30
7.	**I sit because there is nothing to do**	**2.80**
	7. I sit because I have nothing to do	2.40
	11. I sit because it is raining outside	2.70
	17. I sit because there is nothing to do	2.40
	21. I sit because it is too cold outside	2.10
	36. I sit because things go better that way, reading for example	4.40
8.	**I sit because I'm not in the mood to do anything**	**2.60**
	6. I sit because I'm bored	2.40
	32. I sit because I don't feel like standing	2.80

[a] Rated on a 5-point Likert scale with higher scores indicating higher importance
Remaining numbers which were not included in one of the clusters: 39. Because when I feel calm I sit

Table 2. Clusters, statements and importance ratings of children (school 2).

	**Clusters** and statements	Importance[a]
1.	**I sit because I can work better that way/I sit because it is the norm/I sit because I have to**	**3.74**
	3. I sit because I have to at school	4.11
	4. I sit because there is no alternative	3.89
	5. I sit because I can concentrate better this way	4.89
	6. I sit because some activities can only be done seated	3.44
	9. I sit because it is more convenient	3.67
	18. I sit because I have a lot of homework, which I have to do on the computer	3.22
	22. I sit because it is easier	2.67
	24. I sit because I can think better that way	3.67
	31. I sit because writing while standing up is hard	4.11
2.	**I sit because there is nobody to play with**	**3.42**
	2. I sit because there are no other children to play with (in the neighborhood)	3.56
	10. I sit because my parents don't always have the time to play	2.56
	with me	4.00
	19. I sit because it's no fun playing outside on your own	3.11
	23. I sit because classmates live far away, and contact via the phone	
	is easier	3.89
	32. I sit because you need two people to play outside, and it's more fun with two	
3.	**I sit because I'm tired, I want to relax, I want to rest/I sit because**	**3.30**

	**seated activities are fun**	
	1. I sit because I want to rest	3.89
	8. I sit because it is fun	2.89
	12. I sit because it is comfortable to sit on the couch under a blanket	4.00
	14. I sit because standing for too long makes me tired	2.89
	15. I sit because I become calm	3.56
	16. I sit because sometimes I don’t feel like standing	3.33
	17. I sit because I’m tired	3.78
	21. I sit because it doesn’t cost any energy	2.78
	27. I sit because sitting is calmer	2.67
	28. I sit because I don’t want to miss my favorite TV-program	3.78
	29. I sit because sometimes it is more fun	2.78
4.	**I sit because of the weather**	**3.11**
	11. I sit because it is dark outside	2.56
	20. I sit because the weather is bad (cold, rain)	3.67
5.	**I sit because being active takes a lot of effort**	**2.67**
	25. I sit because I have to change my clothes if I want to play	2.44
	outside	2.89
	26. I sit because going outside takes a lot of effort (living on a farm)	

[a] Rated on a 5-point Likert scale with higher scores indicating higher importance
Remaining numbers which were not included in one of the clusters: 7. I sit because it is a habit; 13. I sit because you can use the computer when you are on your own; 30. I sit because my parents tell me to

Table 3. Clusters, statements and importance ratings of children (school 3).

	**Clusters** and statements	Importance[a]
1.	**I sit because it is the norm/I sit because I have to**	**3.69**
	17. I sit because sometimes I have to sit at school	3.88
	18. I sit because my parents tell me to	3.88
	20. I sit because I’m asked to take part in a seated activity, gaming for example	3.38
	23. I sit because it’s easier talking with the family when you’re seated	3.63
2.	**I sit because there is nobody to play with**	**3.69**
	5. I sit because doing something on my own is stupid and I can make contact with the outside world via my phone	3.63
	19. I sit because I can’t arrange to meet people, so then I use the computer to make contact	3.75
3.	**I sit because I’m tired, I want to relax, I want to rest**	**3.46**
	2. I sit because I want to rest	3.38
	13. I sit because it is restful	3.38

	21. I sit because it is relaxed	3.63
4	**I sit because seated activities are fun**	**3.40**
.	11. I sit because I think it is fun	4.00
	14. I sit because sometimes it is more fun to do activities sitting	3.38
	down	3.38
	16. I sit because it is more logical	3.13
	25. I sit because it is fun	3.13
	26. I sit because a seated activity is more interesting	
5	**I sit because there is nothing to do**	**3.31**
.	4. I sit because there are no nice people outside	3.38
	6. I sit because I have nothing to do	3.75
	15. I sit because standing is not convenient	3.13
	28. I sit because I have to wait a long time	3.50
	30. I sit because it is too hot	3.00
	31. I sit because I don't know what to do	3.13
6	**I sit because I'm not in the mood to do anything**	**3.31**
.	1. I sit because I'm tired	3.63
	3. I sit because the weather is bad	3.25
	7. I sit because I don't feel so well (not in the mood)	4.13
	8. I sit because I'm too far from somewhere I can take part in activities (the park)	3.25
	10. I sit because the seated activity is nearby	2.88
	12. I sit because I'm still sleepy	3.38
	22. I sit because I don't feel like doing anything active	3.00
	24. I sit because I'm bored	3.13
	27. I sit because standing for too long makes me tired	3.25
	29. I sit because I already have to do a lot of standing	3.75

[a] Rated on a 5-point Likert scale with higher scores indicating higher importance
Remaining numbers which were not included in one of the clusters: 9. I sit because I feel like it

Table 4. Clusters, statements and importance ratings of children (school 4).

	Clusters and statements	Importance[a]
1.	**I sit because it is the norm/I sit because I have to**	**3.67**
	7. I sit because I'm not allowed to stand while learning in class	2.78
	11. I sit because some activities are impossible to do standing	3.89
	12. I sit because in some activities it is just the way it is done	3.78
	22. I sit because I have to eat	4.00
	37. I sit because it's not possible to stand	3.78
	40. I sit because I have to pray	4.44
	41. I sit because in school I have to	3.44
	42. I sit because sometimes I'm not allowed to stand	3.22

2.	**I sit because I can work better that way**	**3.40**
	14. I sit because standing and running around can be distracting	2.56
	31. I sit because I have to concentrate while I'm working	4.00
	43. I sit because then you can work more accurately	3.44
	49. I sit because sometimes it is more polite	3.67
	50. I sit because some locations are too far to cycle, then I'll go by car	3.33
3.	**I sit because seated activities are fun/I sit because I want to make contact with my friends**	**3.38**
	2. I sit because I like a particular game a lot	3.22
	6. I sit because a friend of mine lives far away, to keep in touch we talk to each other on the phone	4.00
	8. I sit because I play some games online with my friends	3.33
	13. I sit because I can keep in touch with friends online who live far away	3.78
	33. Children sit because they may be addicted to a computer game	2.56
4.	**I sit because of a specific reason**	**3.26**
	32. I sit because when you play hide and seek, people can see you when you stand	3.44
	38. I sit because sometimes being active is dangerous	2.89
	47. I sit because sometimes it is more pleasant for others when you sit	3.44
5.	**I sit because seated activities are fun/I sit because there is nobody to play (actively) with**	**3.13**
	3. I sit because there is nobody to play with	3.56
	9. I sit because when I have to learn I'll do it on my computer	3.44
	21. I sit because I want to watch a program	3.00
	23. I sit because it is cozy to watch YouTube videos	3.44
	29. I sit because my friends want to play inside	2.78
	36. I sit because sometimes I want to laugh at funny videos	3.22
	44. I sit because I want to stay up to date with my social media	2.44
6.	**I sit because I'm tired, I want to relax, I want to rest**	**2.98**
	1. I sit because I'm tired	3.33
	4. I sit when something is bothering me	4.33
	5. I sit when I'm ill	4.00
	10. I sit because I prefer to sit	2.11
	18. I sit because you need less energy for seated activities	2.44
	20. I sit because I'm lazy	2.11
	24. I sit because it is more relaxing	3.67
	25. I sit because it is tiring to stand	1.89
	27. I sit because I'm bored	2.44
	30. I sit because when I'm bored it is an easy solution	3.11
	39. I sit because when you stand for too long you'll get cramps	3.11

	45. I sit because standing activities are tiring	2.22
	46. I sit because I want to rest after a workout	3.78
	51. I sit because sometimes it is more pleasant	3.11
7.	**I sit because the physical environment is not suitable**	**2.75**
	15. I sit because I have to practice in order to be a game programmer when I'm older	2.89
	16. I sit because maybe the weather is bad outside	3.22
	19. I sit because in my neighborhood there are a lot bushes with thorns	2.33
	48. I sit because I love it	2.56

[a] Rated on a 5-point Likert scale with higher scores indicating higher importance
Remaining numbers which were not included in one of the clusters: 17. I sit because I have to practice a lot to be able to play a game well; 26. I sit because I often learn for a good grade; 28. I sit because it is nice and funny; 34. I sit because with my parents I often do activities where I have to sit; 35. I sit because some things are more practical while seated

Table 5. Clusters, statements and importance ratings of parents (purposive sample).

	**Clusters** and statements	Importance[a]
1.	**My child sits because he/she is tired, wants to relax, wants to rest**	**4.10**
	6. Sitting because it is a comfortable posture after some exercise	4.29
	7. Sitting because it is a relaxed posture	4.43
	13. Sitting because he/she wants to rest	4.14
	18. Sitting because he/she then enjoys him/herself	3.43
	23. Sitting because it is a comfortable posture	4.14
	24. Sitting because he/she can then rest	4.14
2.	**My child sits because he/she can work better that way**	**3.88**
	8. Sitting because the activity requires a sitting posture	4.14
	9. Sitting because it is more convenient	3.29
	11. Sitting because it suits the activity	4.29
	14. Sitting because he/she has to travel	3.57
	16. Sitting because he/she has to do homework	3.86
	17. Sitting because the situation demands it	4.00
	20. Sitting because he/she has schoolwork to do	4.14
	22. Sitting because he/she has to study	3.71
3.	**My child sits because there is nothing to do**	**2.62**
	5. Sitting by force of habit	2.43
	12. Sitting because there is nothing active to do	2.14
	15. Sitting to make contact with friends, chatting for example	3.43
	21. Sitting because he/she is bored	2.29

4.	**My child sits because it is the norm**	**2.14**
	1. Sitting because the interior is designed that way	2.00
	2. Sitting because other people in the social environment do it as well	1.71
	10. Sitting because it's what has been taught	2.71

[a] Rated on a 5-point Likert scale with higher scores indicating higher importance
Remaining numbers which were not included in one of the clusters: 3. Sitting because this is the most practical posture for performing the activity; 4. Sitting because he/she has been told to; 19. Sitting because he/she wants to work quietly

Table 6. Clusters, statements and importance ratings of parents (convenience sample)[a].

	**Clusters** and statements	Importance[b]
1.	**My child sits because it is the norm**	**3.52**
	5. Sitting because he/she has to, e.g. at school or while eating/doing homework	4.15
	6. Sitting because he/she is not allowed to lie down	2.46
	7. Sitting because he/she has no other option than to sit or lie down during the activity	4.00
	8. Sitting because there are no desks at school or at home	3.46
	14. Sitting because the activity is designed for a sitting posture, e.g. the computer and TV are placed at sitting height	3.08
	24. Sitting because he/she has been taught to perform the activity in a sitting posture	2.92
	30. Sitting because he/she is expected to sit, e.g. at school, while doing homework or eating	4.23
	31. Sitting because he/she has been told to	3.85
2.	**My child sits because he/she can work/play better that way**	**3.47**
	1. Sitting because the activity requires a sitting posture	4.00
	2. Sitting because it is most appropriate for the activity	3.85
	4. Sitting because it is practical	3.15
	10. Sitting because he/she has to concentrate	3.15
	15. Sitting because he/she can perform the activity more easily that way, eating and doing a puzzle for example	3.15
	16. Sitting because it is a more natural posture for the activity	2.85
	17. Sitting because for some activities there is no other way, e.g. playing on a tablet, watching TV or travelling by car	4.23
	34. Sitting because it is safer to be seated during the activity, playing with a hamster for example	3.38
3.	**My child sits because he/she is tired, wants to rest/relax**	**3.33**
	20. Sitting because it is relaxing	3.62
	21. Sitting because he/she is tired	3.23

	22. Sitting because it is an important relaxation moment	3.62
	35. Sitting because he/she is in a relaxed mood	2.46
	36. Sitting because a seated activity can provide peace and quiet	3.54
	37. Sitting because he/she has to calm down physically	3.85
	41. Sitting because it is a relaxing activity	3.00
4.	**My child sits because seated activities are fun**	**3.32**
	11. Sitting because he/she likes to do seated activities	3.31
	12. Sitting because he/she feels like doing a seated activity	3.00
	13. Sitting because he/she enjoys doing a seated activity	3.54
	18. Sitting because he/she enjoys the activity	4.38
	19. Sitting because he/she likes the seated activity	3.31
	29. Sitting because he/she forgets the world around him/her, while watching TV for example	2.38
5.	**My child sits because its comfortable, and requires less energy**	**3.04**
	3. Sitting because a seated/lying posture is the most comfortable	3.31
	40. Sitting because he/she requires less energy for seated activities	2.77
6.	**My child sits because others do so, and it is a habit**	**2.94**
	9. Sitting because he/she lives in a sedentary society	3.15
	23. Sitting by force of habit	3.08
	25. Sitting because he/she copies sedentary behavior of the parents and others	3.00
	26. Sitting because others nearby are doing seated activities, e.g. watching TV	2.54
7.	**My child sits because it is in his/her nature**	**2.81**
	38. Sitting because he/she is naturally a quiet child	2.38
	39. Sitting because he/she wants to withdraw into him/herself	3.23
8.	**My child sits because there is nothing (active) to do**	**2.52**
	27. Sitting because there is not much to do (that encourages physical activity)	2.54
	28. Sitting because he/she is bored	2.31
	32. Sitting because I (as a parent) don't have the time or don't feel like doing something active with my child	2.23
	33. Sitting because he/she doesn't feel like doing a more active activity, e.g. playing outside	3.00
	42. Sitting because some seated activities easily fill up the day, he/she doesn't have to think about what he/she can do, e.g.	2.46
	watching TV	2.15
	43. Sitting because he/she can't think of anything else to do	2.92
	44. Sitting because he/she has nobody to play with	

[a] Sorting task n=14, rating task n=13

[b] Rated on a 5-point Likert scale with higher scores indicating higher importance
