# Supplementary material for: Why Do Children Engage in Sedentary Behavior? Child- and Parent-Perceived Determinants
Source: Int J Environ Res Public Health. 2017 Jun 22;14(7):671. doi: 10.3390/ijerph14070671 (PMC5551109; doi:10.3390/ijerph14070671)
Supplement: Supplementary File 1 [file ijerph-14-00671-s001.zip › ijerph-195872-S3.pdf]

Appendix S3 - Concept maps children (school 1, 3 and 4) and parents (purposive sample)

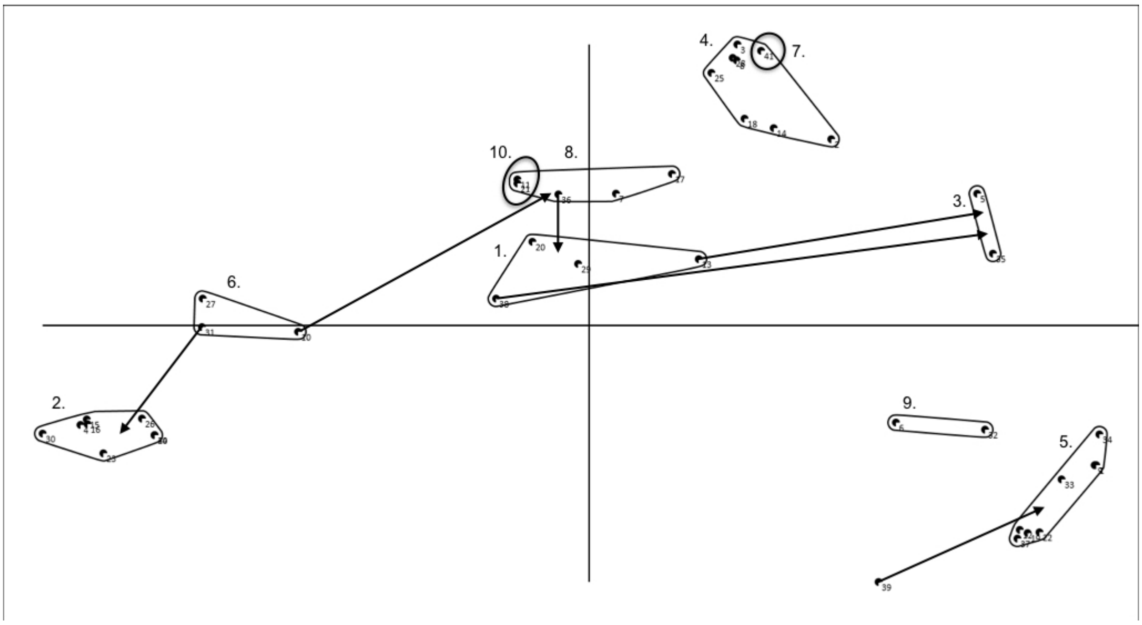

Figure 1. Concept map children (school 1).

Note that points, i.e. statements, sorted more often together appear closer to each other, and statements never/rarely sorted together appear widely separated.

Arrows indicate a statement is reallocated by researchers, circles indicate a new cluster is created by researchers as a result of reallocation of statements.

<u>Cluster 1</u>: I sit because I can work/play better that way; <u>Cluster 2</u>: I sit because it is the norm/I sit because I have to; <u>Cluster 3</u>: I sit because it feels better; <u>Cluster 4</u>: I sit because seated activities are fun; <u>Cluster 5</u>: I sit because I'm tired, I want to relax, I want to rest; <u>Cluster 6</u>: I sit because of my health; <u>Cluster 7</u>: I sit because there is nobody to play with; <u>Cluster 8</u>: I sit because there is nothing to do; <u>Cluster 9</u>: I sit because I'm not in the mood to do anything; <u>Cluster 10</u>: I sit because of the weather

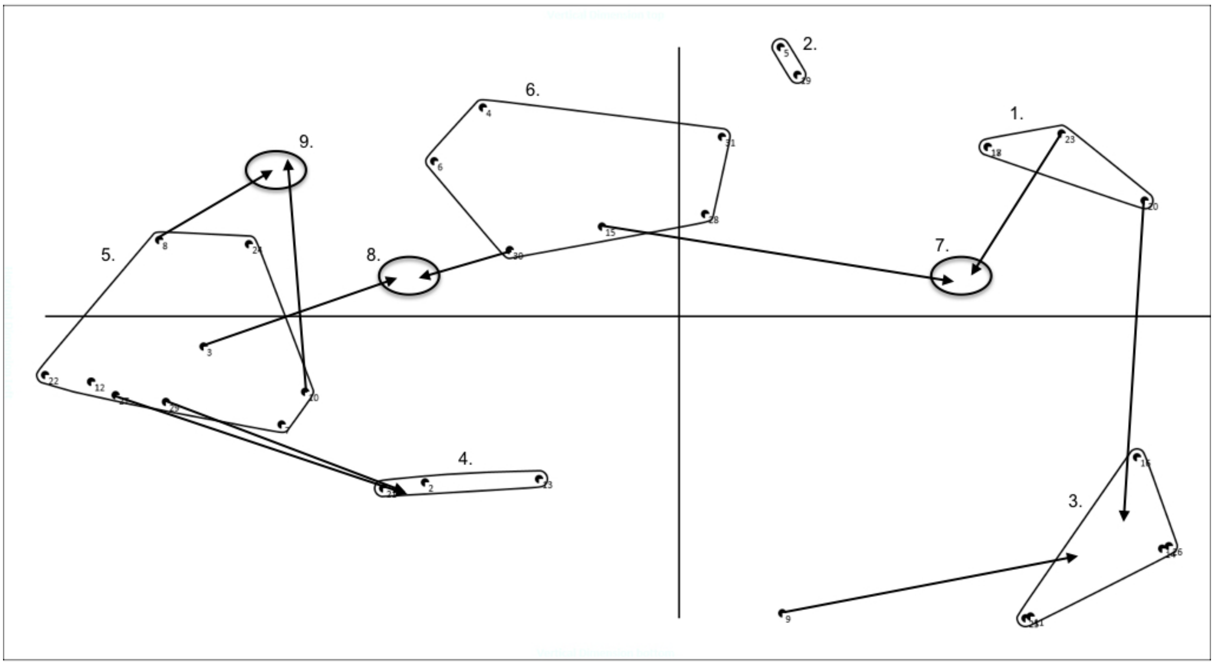

Figure 2. Concept map children (school 3).

Note that points, i.e. statements, sorted more often together appear closer to each other, and statements never/rarely sorted together appear widely separated.

Arrows indicate a statement is reallocated by researchers, circles indicate a new cluster is created by researchers as a result of reallocation of statements.

Cluster 1: I sit because it is the norm/I sit because I have to; Cluster 2: I sit because there is nobody to play with; Cluster 3: I sit because seated activities are fun; Cluster 4: I sit because I'm tired, I want to relax, I want to rest; Cluster 5: I sit because I'm not in the mood to do anything; Cluster 6: I sit because there is nothing to do; Cluster 7: I sit because this posture suits the activity better; Cluster 8: I sit because of the weather; Cluster 9: I sit because the physical environment suitable for physical activities is too far away

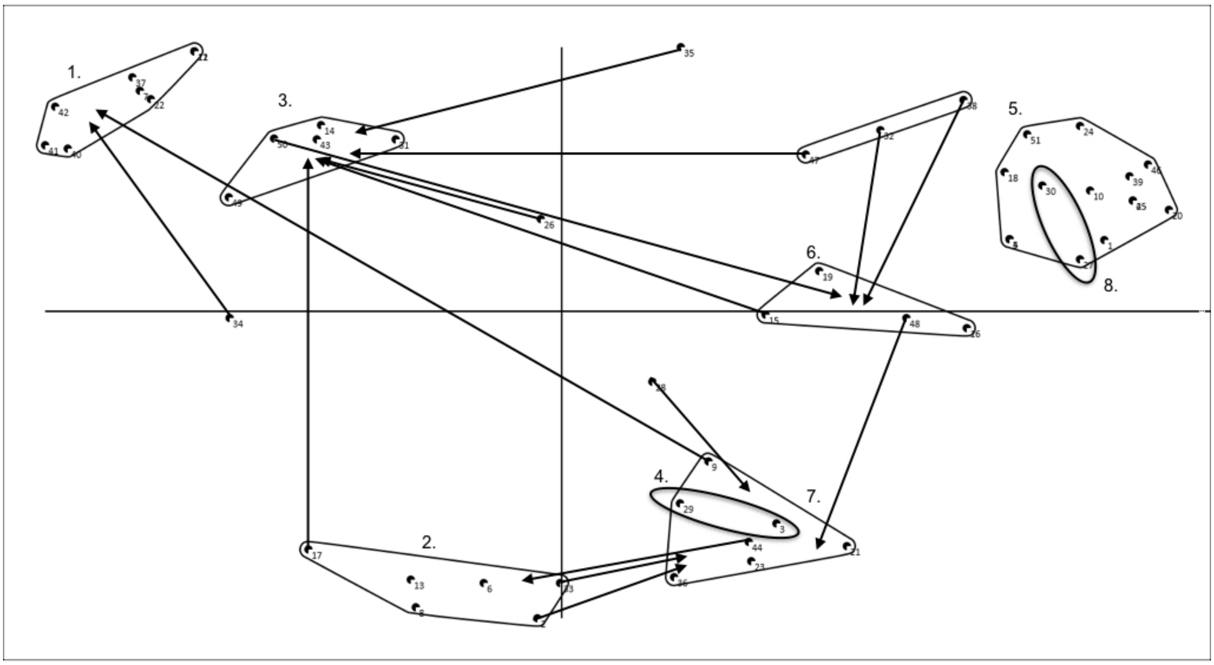

Figure 3. Concept map children (school 4).

Note that points, i.e. statements, sorted more often together appear closer to each other, and statements never/rarely sorted together appear widely separated.

Arrows indicate a statement is reallocated by researchers, circles indicate a new cluster is created by researchers as a result of reallocation of statements.

<u>Cluster 1</u>: I sit because it is the norm/I sit because I have to; <u>Cluster 2</u>: I sit because I want to make contact with my friends; <u>Cluster 3</u>: I sit because I can work/play better that way; <u>Cluster 4</u>: I sit because there is nobody to play (actively) with; <u>Cluster 5</u>: I sit because I'm tired, I want to relax, I want to rest; <u>Cluster 6</u>: I sit because the physical environment is not suitable; <u>Cluster 7:</u> I sit because seated activities are fun; <u>Cluster 8:</u> I sit because there is nothing to do

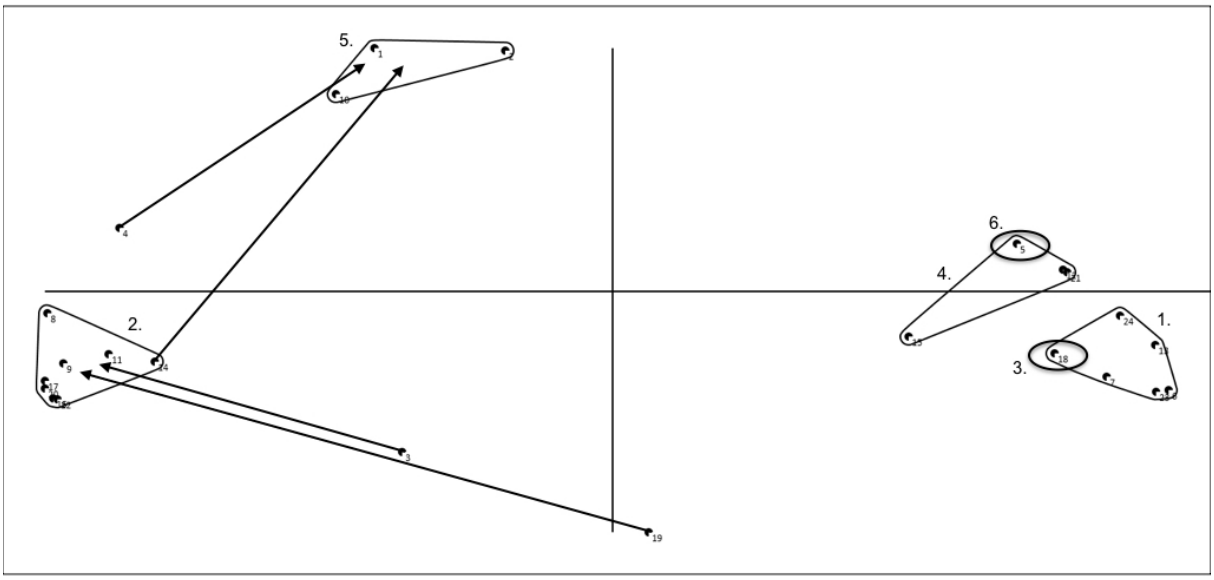

Figure 4. Concept map parents (purposive sample).

Note that points, i.e. statements, sorted more often together appear closer to each other, and statements never/rarely sorted together appear widely separated.

Arrows indicate a statement is reallocated by researchers, circles indicate a new cluster is created by researchers as a result of reallocation of statements.

<u>Cluster 1</u>: My child sits because he/she it tired, wants to relax, wants to rest; <u>Cluster 2</u>: My child sits because he/she can work/play better that way; <u>Cluster 3</u>: My child sits because seated activities are fun; <u>Cluster 4</u>: My child sits because there is nothing to do; <u>Cluster 5</u>: My child sits because it is the norm; <u>Cluster 6</u>: My child sits because it is a habit
